# SG-SP1 Suppresses Mast Cell-Mediated Allergic Inflammation via Inhibition of FcεRI Signaling

**DOI:** 10.3389/fimmu.2020.00050

**Published:** 2020-01-28

**Authors:** Min-Jong Kim, In-Gyu Je, Jaeyoung Song, Xiang Fei, Soyoung Lee, Huiseon Yang, Wonku Kang, Yong Hyun Jang, Seung-Yong Seo, Sang-Hyun Kim

**Affiliations:** ^1^CMRI, Department of Pharmacology, School of Medicine, Kyungpook National University, Daegu, South Korea; ^2^Research Laboratories, ILDONG Pharmaceutical Co. Ltd., Hwaseong, South Korea; ^3^New Drug Development Center, Daegu-Gyeongbuk Medical Innovation Foundation, Daegu, South Korea; ^4^College of Pharmacy, Gachon University, Incheon, South Korea; ^5^Immunoregulatory Materials Research Center, Korea Research Institute of Bioscience and Biotechnology, Jeongeup, South Korea; ^6^College of Pharmacy, Chung-Ang University, Seoul, South Korea; ^7^Department of Dermatology, School of Medicine, Kyungpook National University, Daegu, South Korea

**Keywords:** SG-SP1, gallic acid, mast cells, allergic inflammation, FcεRI

## Abstract

**Background:** As the number of allergic disease increases, studies to identify new treatments take on new urgency. Epigallocatechin gallate (EGCG), a major component of green tea, has been shown to possess a wide range of pharmacological properties, including anti-inflammation and anti-viral infection. In previous study, gallic acid (GA), a part of EGCG, has shown anti-allergic inflammatory effect. To improve on preliminary evidence that GA has allergy mitigating effect, we designed SG-SP1 based on GA, and aimed to assess the effects of SG-SP1 on mast cell-mediated allergic inflammation using various animal and *in vitro* models.

**Methods:** For *in vitro* experiments, various types of IgE-stimulated mast cells (RBL-2H3: mast cell-like basophilic leukemia cells, and primary cultured peritoneal and bone marrow-derived mast cells) were used to determine the role of SG-SP1 (0.1–1 nM). Immunoglobulin (Ig) E-induced passive cutaneous anaphylaxis and ovalbumin-induced systemic anaphylaxis, standard animal models for immediate-type hypersensitivity were also used.

**Results:** For *in vitro*, SG-SP1 reduced degranulation of mast cells by down-regulating intracellular calcium levels in a concentration-dependent manner. SG-SP1 decreased expression and secretion of inflammatory cytokines in activated mast cells. This suppressive effect was associated with inhibition of the phosphorylation of Lyn, Syk and Akt, and the nuclear translocation of nuclear factor-κB. Due to the strong inhibitory effect of SG-SP1 on Lyn, the known upstream signaling to FcεRI-dependent pathway, we confirmed the direct binding of SG-SP1 to FcεRI, a high affinity IgE receptor by surface plasmon resonance experiment. Oral administration of SG-SP1 hindered allergic symptoms of both anaphylaxis models evidenced by reduction of hypothermia, serum IgE, ear thickness, and tissue pigmentation. This inhibition was mediated by the reductions in serum histamine and interleukin-4.

**Conclusions:** We determined that SG-SP1 directly interacts with FcεRI and propose SG-SP1 as a therapeutic candidate for mast cell-mediated allergic inflammatory disorders *via* inhibition of FcεRI signaling.

## Introduction

Allergic disorders including atopic dermatitis, allergic rhinitis, asthma, food allergies, and anaphylaxis are prominent in the modern world (1). The prevalence of allergic disorders in developed countries dramatically increasing from the last decade (2). Mast cells are key players in allergic inflammation and act through the production and secretion of allergic mediators such as histamine, chemokines, cytokines, and platelet activating factor (3, 4). Histamine, a major factor of acute allergic responses, induces hypothermia and recruitment of leukocytes following vasodilation and an increase in vascular permeability. Inflammatory cytokines including tumor necrosis factor (TNF)-α, interleukin (IL)-1β, IL-4, and IL-6 support a chronic phase of allergy, enhancing T cell activation or B cell survival (4). Therefore, therapeutic strategies such as glucocorticoids, anti-histamines, and non-steroidal anti-inflammatory drugs (NSAIDs) targeted mast cells to suppress allergic diseases including rhinitis and asthma (5–7).

Activation of mast cells is initiated by allergen-induced cross-linking of IgE bound by the FcεRI, a high affinity IgE receptor, on the surface of cells. FcεRI is a heterotetrameric receptor comprised of an IgE-binding α subunit, the membrane tetraspanning β subunit, and two identical disulphide linked γ subunits (4). Assembly of the heterotetrameric structure of FcεRI is initiated by the phosphorylation of the immunoreceptor tyrosine-based activating motifs on the β and γ subunits by Src family kinases such as Lyn and Fyn (8, 9). Lyn and Syk phosphorylate several adaptor molecules and enzymes to regulate mast cell activation. In addition to Lyn, FcεRI aggregation activates Fyn, which phosphorylates the adaptor Gab2 to activate the PI3K pathway (4). Therefore, it would be effective to inhibit mast cell activation by binding directly to FcεRI. To confirm the direct binding, surface plasmon resonance (SPR) has been used as an experimental technique. As this assay measured biomolecular interactions in a real-time manner, SPR binding analysis is mainly used to study molecular interactions (4).

We previously showed that gallic acid (GA) and its derivatives inhibited mast cell-derived allergic inflammatory reactions by blocking histamine release and pro-inflammatory cytokine expression (10, 11). Despite the inhibitory activity of GA on allergic inflammation, we were intrigued to design new derivatives of GA to strengthen the pharmacological effectiveness of GA. Amide and ester derivatives based on a GA scaffold for which substituent carboxylic acids and three hydroxyl groups of C3, C4, and C5 positions could be selected. Our medicinal chemistry-based efforts to modify phenol groups as well as form amides into a proper amine substituent led to the identification of compound SG-SP1, which consists of 3,4,5-trisbenzyloxyphenyl and (*S*)-phenylglycinyl groups and acts as a potent inhibitor of mast cell-mediated allergic inflammation. In the present study, we examined the anti-allergic inflammatory properties of SG-SP1 using immediate-type hypersensitivity models and elucidated the underlying inhibitory mechanism of SG-SP1 binding directly to FcεRI in mast cells.

## Materials and Methods

### General Procedure

All starting materials and reagents were obtained from commercial suppliers and were used without further purification. Air- and moisture-sensitive reactions were performed under an argon atmosphere. Flash column chromatography was performed with silica gel 60 (230–400 mesh, Merck KGaA, Darmstadt, Germany) and the indicated solvents. Thin-layer chromatography was performed using 0.25 mm silica gel plates (Merck KGaA). ^1^H- and ^13^C-NMR spectra were recorded on a Bruker 600 MHz spectrometer with solutions of deuteriochloroform (CDCl_3_) or methanol-d4 (Cambridge Isotope Laboratory, Andover, MA). ^1^H-NMR data were reported as the order of chemical shift, multiplicity (s, singlet; d, doublet; t, triplet; m, multiplet; and/or multiple resonances), number of protons, and coupling constant (J) in hertz (Hz). High-resolution mass spectra (HRMS) were recorded on a JEOL JMS-700 (EI) (JEOL Ltd., Tokyo, Japan) and Agilent 6530 Q-TOF LC/MS/MS system (ESI) (Agilent Technologies, Inc., Santa Clara, CA).

### Synthesis of SG-SP1

For SG-SP1, a DMF solution (2 mL) of 3,4,5-tris(benzyloxy)benzoic acid (110 mg, 0.25 mmol) was supplemented with (S)-2-phenylglycine methyl ester hydrochloride (56 mg, 0.28 mmol), EDCI (115 mg, 0.60 mmol), HOBt (4.0 mg, 30 μmol) and N, N-diisopropylethylamine (90 μL, 0.50 mmol). After stirring for 3 h, the reaction mixture was diluted with ethyl acetate and washed with water and brine, dried over MgSO_4_, and concentrated on reduced pressure. The residue was purified by flash column chromatography on a silica gel (ethyl acetate/*n*-hexane = 1:3) to produce the desired amide. Compound 3d was identified as SG-SP1 (12).

All drug including SG-SP1, GA, and dexamethasone (Dexa) were dissolved in dimethyl sulfoxide (DMSO) and then diluted with phosphate-buffered saline (PBS). The percentage DMSO in the treated samples was maintained at <0.5% to minimize the interference.

### Animals

Imprinting Control Region (ICR) mice (male, 6 weeks, 22–25 g) and Sprague-Dawley (SD) rats (male, 10 weeks, 250–280 g) were purchased from Dae-Han Biolink (Daejeon, Republic of Korea). In many clinical studies, the incidence of systemic anaphylaxis has been shown that women are more likely to have anaphylaxis than men (13). Therefore, female animals are preferred. However, due to the size of local tissue (ear), male animals were used in this study. Throughout the study, five animals per cage were housed in a room with laminar air flow, a temperature of 22 ± 2°C, and relative humidity of 55 ± 5%. Animal care and treatments were carried out in accordance with the guidelines established by the Public Health Service Policy on the Humane Care and Use of Laboratory Animals and were approved by the Institutional Animal Care and Use Committee of Kyungpook National University (IRB #2017-0002-9).

### Reagents and Cell Culture

Dexamethasone (Dexa), anti-dinitrophenyl (DNP) IgE, DNP-human serum albumin (HSA), ovalbumin (OVA), and Histodenz were purchased from Sigma-Aldrich (St. Louis, MO), and alum adjuvant was purchased from Thermo Fisher Scientific (Waltham, MA). Rat basophilic leukemia (RBL-2H3) cells purchased from Korean Cell Line Bank (Seoul, Republic of Korea). RBL-2H3, rat peritoneal mast cells (RPMC), and mouse bone marrow-derived mast cells (mBMMC) were grown at 37°C in 5% CO_2_ in Dulbecco's modified Eagle's medium, α-minimum essential medium (Gibco, Grand Island, NY) and RPMI 1640 (Hyclone, Logan, UT), respectively, supplemented with 10% heat-inactivated fetal bovine serum, 100 U/mL penicillin G, 100 μg/mL streptomycin, and 250 ng/mL amphotericin B. In addition, complete RPMI 1640 media was supplemented by 4 mM L-glutamine, 25 M HEPES, 50 μM 2-mercaptoethanol, 1 mM sodium pyruvate, MEM non-essential amino acid solution (Gibco), 10 ng/mL murine IL-3, and 2 ng/mL murine SCF (PeproTech, Rocky Hill, NJ). RBL-2H3 were used throughout the study at passages ranging from 4 to 8.

### Preparation of mBMMCs

Mouse bone marrow-derived mast cells (mBMMCs) were isolated from male ICR mouse as previously described (14). All subsequent steps were performed into the sterile cell culture hood to prevent contamination of the bone marrow. The isolated cells were grown in complete RPMI 1640 and supplemented with recombinant murine IL-3 (10 ng/mL) and stem cell factor (SCF, 2 ng/mL) (PeproTech) for maturation during 3 weeks. After maturation, cells were seeded on the culture plates without IL-3 and SCF, sensitized with anti-DNP IgE for overnight, and then stimulated with DNP-HSA.

### Preparation of RPMCs

Rat peritoneal mast cells (RPMC) were isolated from SD rats as previously described (10). The peritoneal cavity was carefully opened, and the fluid containing peritoneal cells was aspirated using a Pasteur pipette. An abdomen was massaged during washing the intestines with fluid. The mast cell preparations were approximately 95% pure, based on toluidine blue staining, and more than 97% of the cells were viable, based on trypan blue staining.

### Determination of Cell Viability

Cell viability was determined by colorimetric analysis using 3-(4,5-dimethylthiazol-2-yl)-2,5-diphenyltetrazolium bromide (MTT) (15). Water-soluble MTT is converted into water-insoluble formazan by mitochondrial dehydrogenase. RBL-2H3 (3 × 10^4^/well in 96-well plates) were pretreated with various concentrations of SG-SP1 for 24 h and incubated with 1 mg/mL MTT at 37°C. After 2 h, the formazan crystals were dissolved with DMSO, and then the absorbance was measured using a spectrophotometry (Molecular Devices, Sunnyvale, CA) at a wavelength of 570 nm. The formazan formed absorbance of untreated control was assigned as a relative value of 100%.

In order to further to analyse the cell viability, cell death was detected by Annexin V and propidium iodide (PI) staining. Before staining, RBL-2H3 (5 × 10^5^/well in 12-well plates) were pretreated with various concentrations of SG-SP1 for 24 h. The cells were then washed with PBS, centrifuged, and resuspended in binding buffer (10 mM HEPES, 140 mM NaCl, and 2.5 mM CaCl_2_, pH 7.4) containing 5 μg/mL of Annexin V and PI following the manufacturer's protocol. The cells were incubated at 37°C in 5% CO_2_ for 20 min and analyzed using a flow cytometer (BD Biosciences, San Diego, CA). The fluorochrome was excited using the 488 nm line of an argon ion laser, and emissions of Annexin V and PI were monitored at 525 and 620 nm. A total of at least 1 × 10^4^ cells was analyzed per each sample. The relative cell events of untreated control were assigned as a relative value of 1.

### Determination of Mast Cell Degranulation

To determine mast cell degranulation, levels of histamine in sera and culture media were measured. Mouse blood was centrifuged at 400 g for 15 min at 4°C, and sera were obtained. RBL-2H3 (5 × 10^5^/well in 12-well plates) were sensitized with anti-DNP IgE (50 ng/mL). After incubation overnight, cells were pretreated with or without drugs for 1 h and then challenged on DNP-HSA (100 ng/mL) for 4 h. mBMMCs (5 × 10^5^/well in 12-well plates) and RPMCs (2 × 10^4^/well in 24-well plates) were subjected to that same treatment as RBL-2H3; however, these were challenged on DNP-HSA for 30 min. The cells were separated from the media by centrifugation at 150 *g* for 5 min at 4°C. To separate histamine from sera and media, 0.1 N HCl and 60% perchloric acid were added. After centrifugation, supernatants were transferred to Eppendorf tubes containing 5 N NaOH, 5 M NaCl, and *n*-butanol, and then vortexed. The organic phase was gathered, mixed with 0.1 N HCl and *n*-heptane, and centrifuged. The histamine in the aqueous phase was assayed using an *o*-phthaldialdehyde spectrofluorometric procedure as previously described (16). Fluorescence intensity was measured using a fluorescent plate reader (Molecular Devices) at an excitation wavelength of 360 nm and an emission wavelength of 440 nm. Release of β-hexosaminidase is also widely used as a marker for mast cell degranulation (16). After incubation of media with β-hexosaminidase substrate buffer (100 mM sodium citrate, 1 mM 4-nitrophenyl *N*-acetyl-β-D-galactosaminide, pH 4.5) for 1 h at 37°C, the reaction was terminated using a stop solution (0.1 M Na_2_CO_3_ and NaHCO_3_), and the absorbance was measured using a spectrophotometry at a wavelength of 405 nm. The percentage of β-hexosaminidase release was calculated from the equation: [β-hexosaminidase release (%) = (absorbance of supernatant) / (absorbance of supernatant + absorbance of pellet) × 100].

### RNA Extraction and Quantitative Real-Time PCR (qPCR)

Prior to isolation from total cellular RNA, RBL-2H3 and mBMMC (5 × 10^5^/well in 12-well plates) were sensitized with anti-DNP IgE (50 ng/mL). After incubation overnight, the cells were pretreated with or without drugs for 1 h and challenged with DNP-HSA (100 ng/mL) for 1 h. RNAiso Plus reagent (Takara Bio Inc., Shiga, Japan) was used to extract total RNA in accordance with the manufacturer's protocol. Complementary DNA (cDNA) was synthesized from 2 μg of total RNA using a Maxime RT-Pre Mix Kit (iNtRON Biotechnology, Daejeon, Republic of Korea). qPCR was carried out using a Thermal Cycler Dice TP850 (Takara Bio Inc.) according to the manufacturer's protocol. The total 25 μL reaction mixture was composed of the following: 1.5 μL of cDNA (150 ng), 1 μL each of the forward and reverse primers (0.4 μM), 12.5 μL of SYBR Premix Ex Taq (Takara Bio Inc.), and 9 μL of dH_2_O. The conditions used for PCR were the same as those described previously (8). The sequences of primers are listed in Table S1. The gene expression ratio of untreated control was assigned as a relative value of 1.

### Determination of Intracellular Calcium Levels

Intracellular calcium was measured using the fluorescent indicator Fluo-3/AM (Invitrogen, Carlsbad, CA) (10). RBL-2H3 and mBMMC (6 × 10^4^/well in 96-well plates) were sensitized with anti-DNP IgE (50 ng/mL). After incubation overnight, the cells were preincubated with Fluo-3/AM for 1 h at 37°C and washed with Tyrode's buffer B (137 mM NaCl, 5.5 mM glucose, 12 mM NaHCO_3_, 2.7 mM KCl, 0.2 mM NaH_2_PO_4_, 1 mM MgCl_2_, and 1.8 mM CaCl_2_) to remove the dye from the cell surface. The cells were pretreated with or without drugs including SG-SP1, GA, and BAPTA-AM (Calbiochem, La Jolla, CA) for 1 h and then challenged on DNP-HSA (100 ng/mL). BAPTA-AM, a calcium chelator, was used as a positive control. Fluorescence intensity was detected using a fluorescent plate reader at an excitation wavelength of 485 nm and an emission wavelength of 510 nm. The intracellular calcium level of untreated control was assigned as a relative value of 1.

### ELISA

The levels of inflammatory cytokines in sera and culture media were measured by ELISA (8). RBL-2H3 (5 × 10^5^/well in 12-well plates) were sensitized with anti-DNP IgE (50 ng/mL). After incubation overnight, the cells were pretreated with or without drugs for 1 h and then challenged with DNP-HSA (100 ng/mL) for 8 h. The cells were separated from media by centrifugation at 150 *g* for 5 min at 4°C. ELISA was performed on a 96-well Nunc immune plate using a commercial kit (BD Biosciences, San Diego, CA) according to the manufacturer's protocol. Before detection of OVA-specific IgE, immune plates were coated with 20 μg of OVA instead of capture antibody. After terminating the reaction to a substrate, the absorbance was measured using a spectrophotometry at a wavelength of 450 nm. The cytokine secretion ratio of untreated control was assigned as a relative value of 1.

### Protein Extraction and Western Blot

Nuclear and cytoplasmic proteins were extracted as previously described (16). Before protein extraction, RBL-2H3 (2 × 10^6^/well in 6-well plates) were sensitized with anti-DNP IgE (50 ng/mL). After incubation overnight, cells were pretreated with or without drugs for 1 h and challenged on DNP-HSA (100 ng/mL). After suspension in 100 μL of cell lysis buffer A (0.5% Triton X-100, 150 mM NaCl, 10 mM HEPES, 1 mM EDTA/Na_3_VO_4_, 0.5 mM PMSF/DTT, and 5 μg/mL leupeptin/aprotinin), the cells were vortexed, incubated for 5 min on ice, and centrifuged at 400 g for 5 min at 4°C. The supernatant was collected and used as the cytoplasmic protein extract. The pellets were washed three times with 1 mL of PBS and then suspended in 25 μL of cell lysis buffer B (25% glycerol, 420 mM NaCl, 20 mM HEPES, 1.2 mM MgCl_2_, 0.2 mM EDTA, 1 mM Na_3_VO_4_, 0.5 mM PMSF/DTT, and 5 μg/mL leupeptin/aprotinin), vortexed, sonicated for 30 s, incubated for 20 min on ice, and centrifuged at 15,000 g for 15 min at 4°C. The supernatant was collected and used as the nuclear protein extract. Proteins were separated by 8–12% SDS-PAGE and transferred to a nitrocellulose membrane. Immunodetection was carried out using a chemiluminescent substrate (Thermo Fisher Scientific). The protein production ratio of untreated control was assigned as a relative value of 1. The following antibodies were purchased from Santa Cruz Biotech (Santa Cruz, CA); NF-κB (sc-109), IκBα (sc-371), lamin B1 (sc-374015), and β-actin (sc-8432). The following antibodies were purchased from Cell Signaling Technology (Beverly, MA); phospho-Lyn (#2731, Tyr^507^), Lyn (#2732), phospho-Syk (#2711, Tyr^525/526^), Syk (#2712), phospho-Akt (#9271, Ser^473^), and Akt (#9272). The following antibodies were purchased from Abcam (Cambridge, UK); phospho-Fyn (ab182661, Tyr^530^), and Fyn (ab125016).

### SPR Binding Analysis

Physical interactions between compounds and FcεRIα were analyzed by SPR experiment using a Biacore T200 instrument (GE Healthcare Life Sciences, Chicago, IL) FcεRIα was immobilized on carboxylmethyl-dextran sensor chip (CM) by the amine-coupling method. FcεRIα was diluted in 30 μg/mL HBS-EP buffer (GE Healthcare) and injected into a rate of 5 μL/mL for tandem immobilization on the CM5 chip surface, resulting in from 1,300 response units after stabilization. Compounds (100 μM) were prepared by dilution in HBS-EP buffer at the 1% DMSO and injected into the FcεRIα protein-coated flow channel at a flow of 30 μL/mL, followed by a dissociation time for 300 s and a regeneration with 50 mM NaOH. Raw sensorgrams were double blanked by subtracting responses from reference flow channel, a blank injection, using BiaEvaluation Software (GE Healthcare). All SPR experiments were performed at 20°C.

### IgE-Mediated Passive Cutaneous Anaphylaxis

An IgE-mediated passive cutaneous anaphylaxis (PCA) model was established as described previously (8). To induce the PCA reaction, mice were randomly divided into 7 groups (*n* = 5/group), and the skin on the ears of mice was sensitized with an intradermal (i.d.) injection of anti-DNP IgE (0.5 μg/site) for 48 h. Drugs were orally administered at doses of 0.1–10 mg/kg body weight 2 h before intravenous (i.v.) injection of DNP-HSA (1 mg/mouse) and 4% Evans blue (1:1) mixture. Thirty minutes later, the mice were euthanized, and both ears were collected to measure dye pigmentation. The amount of dye was determined colorimetrically after extraction with 1 mL of 1 M KOH and 4 mL of an acetone and phosphoric acid (5:13) mixture. The absorbance of each extract was measured using a spectrophotometry at a wavelength of 620 nm.

### OVA-Induced Systemic Anaphylaxis

Mice were randomly divided into 5 groups (*n* = 10/group) and sensitized with an OVA mixture (100 μg of OVA and 2 mg of alum adjuvant in 200 μL of PBS) by intraperitoneal (i.p.) injection on day 0 and 7. Drugs including SG-SP1, GA, and Dexa were orally administered three times at doses of 0.1-10 mg/kg body weight once every 2 days after the second sensitization. On day 14, 200 μg of OVA in 100 μL of PBS was i.p. injected, and then rectal temperatures were measured every 10 min for 1 h. After 1 h, blood was obtained from the abdominal vein of each mouse to measure serum histamine, OVA-specific IgE, and IL-4 levels.

### Statistical Analysis

Statistical analyses were performed using Prism 5 (GraphPad Software, San Diego, CA), and treatment effects were analyzed using a one-way analysis of variance followed by Dunnett's test. A value of *p* < 0.05 was considered a significant difference.

## Results

### Effects of SG-SP1 on the Degranulation of Mast Cells and Expression of Inflammatory Cytokines

**Figure 1:**
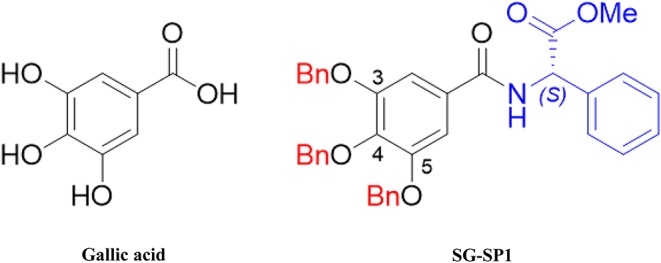
Chemical structures of gallic acid and SG-SP1. Bn, benzoyl ether.

The chemical structures of gallic acid (GA) and SG-SP1 were displayed in Figure 1. To rule out cytotoxic influences of SG-SP1, MTT assay and Annexin V/PI staining were carried out. Up to 100 μM of SG-SP1 resulted in no significant cytotoxicity after 24 h exposure to RBL-2H3 (Figure S1).

**Figure 2:**
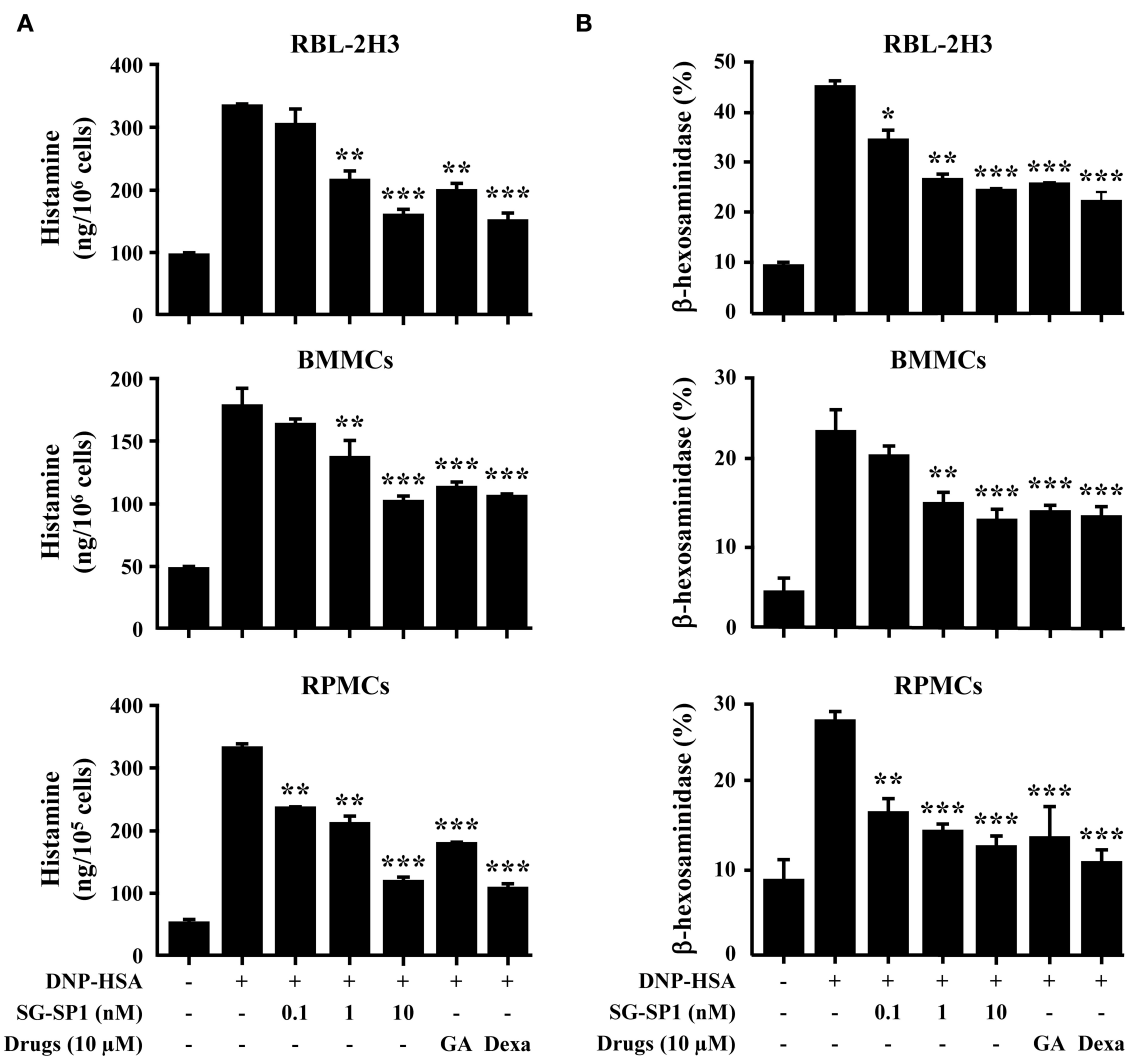
Effects of SG-SP1 on mast cell degranulation. **(A)** RBL-2H3, mBMMCs (5 × 10^5^/well), and RPMCs (2 × 10^4^/well) were sensitized with anti-DNP IgE (50 ng/mL). After incubation overnight, the cells were pretreated with or without drugs including SG-SP1, GA, and Dexa for 1 h and then challenged with DNP-HSA (100 ng/mL). Histamine levels were assayed using an *o*-phthaldialdehyde spectrofluorometric procedure. **(B)** The level of β-hexosaminidase was measured using β-hexosaminidase substrate buffer. Data were presented as the means ± SEM of three independent experiments.*, **, and *** indicate statistically significant differences from the DNP-HSA-challenged group at a value of *p* < 0.05, 0.01, and 0.001, respectively.

Mast cells are important sources of histamine, which plays a key role in allergic symptoms (17). Attenuation of mast cell degranulation could be a therapeutic strategy to regulate allergic responses. We demonstrated the influence of SG-SP1 on degranulation of mast cells using RBL-2H3 (mast cell-like basophilic leukemia cells) and primary cultured mast cells (RPMCs and mBMMCs). Histamine release was rapidly induced by antigen challenge and concentration-dependently suppressed by SG-SP1 treatment in all three types of mast cells. β-Hexosaminidase, another component in the mast cell granule, is also used to assay mast cell degranulation (16, 18). SG-SP1 similarly reduced levels of β-hexosaminidase as well as histamine (Figure 2).

**Figure 3:**
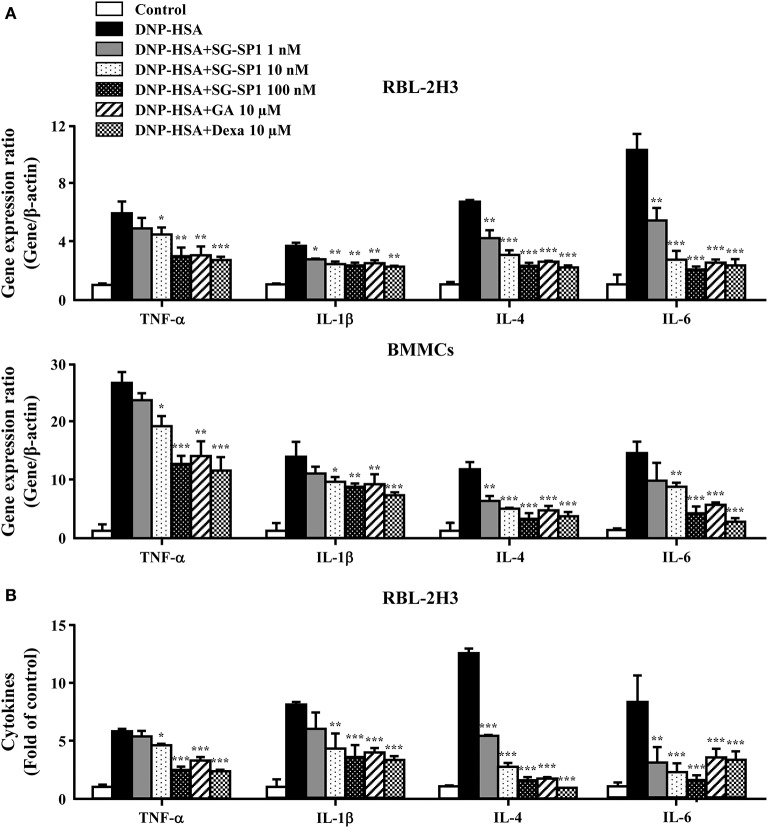
Effects of SG-SP1 on inflammatory cytokine expression. RBL-2H3 and BMMCs were sensitized with anti-DNP IgE. After incubation overnight, the cells were pretreated with or without drugs including SG-SP1, GA, and Dexa for 1 h and then challenged with DNP-HSA. **(A)** The expression of inflammatory cytokine genes was determined by qPCR. **(B)** The secretion of inflammatory cytokines was measured by ELISA. Data were presented as the means ± SEM of three independent experiments. *, **, and *** indicate statistically significant differences from the DNP-HSA-challenged group at a value of *p* < 0.05, 0.01, and 0.001, respectively.

Inflammatory cytokines, especially IL-4, are highly associated with allergic inflammation because of their recruitment and activation of immune cells (19). To ascertain the effects of SG-SP1 on the expression of inflammatory cytokines such as TNF-α, IL-1β, IL-4 and IL-6 in RBL-2H3 and BMMCs, qPCR and ELISA were performed. The expression of these cytokines at both the mRNA and protein levels were elevated by activation of FcεRI, whereas it was concentration-dependently suppressed by SG-SP1 (Figure 3). For evaluation of qPCR results, we used two reference genes, β-actin and GAPDH. Gene expression ratio with two reference genes showed similar results and we showed ratio with β-actin (Figure 3A).

### Effects of SG-SP1 on Intracellular Calcium Levels in Mast Cells

**Figure 4:**
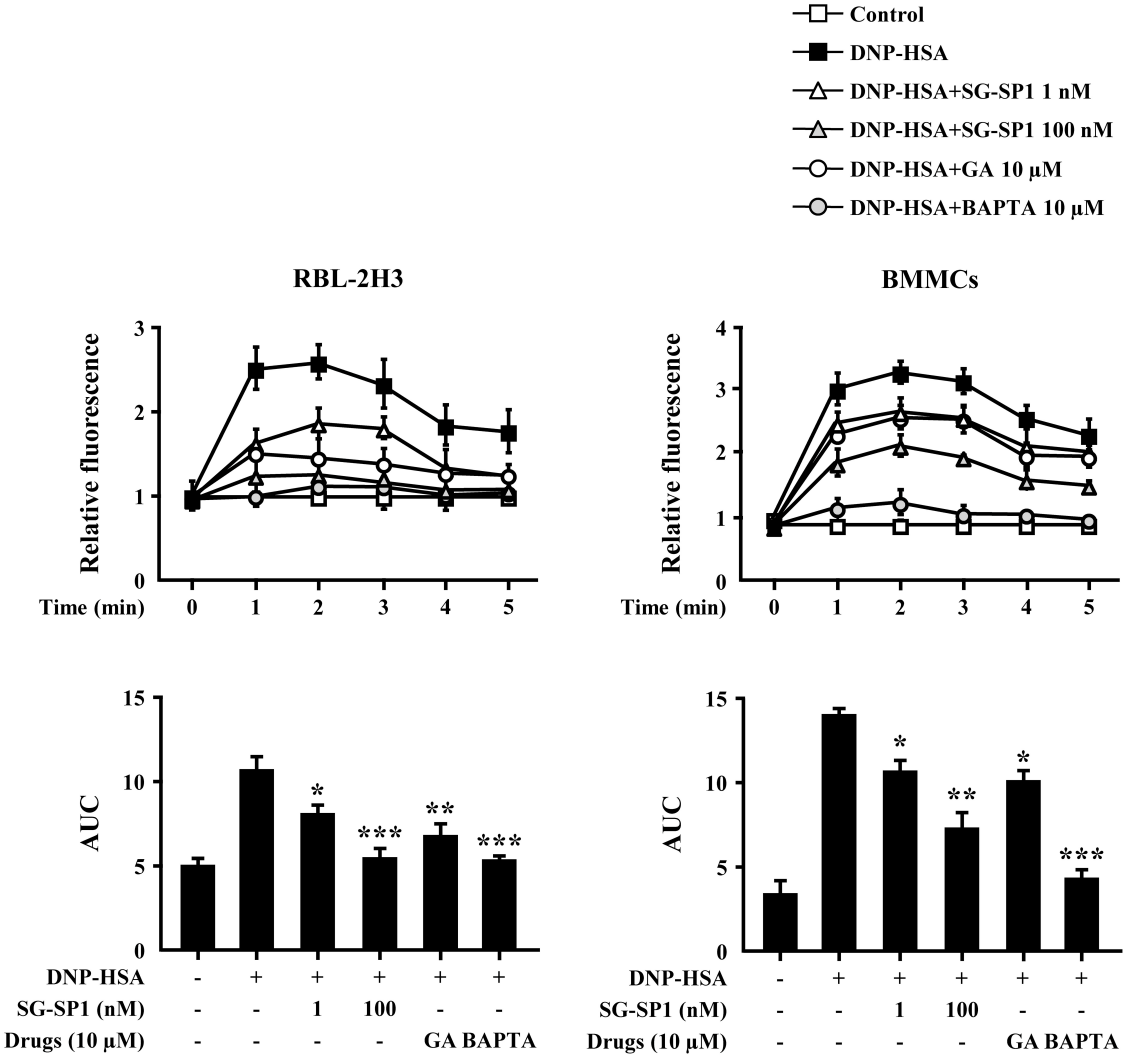
Effects of SG-SP1 on intracellular calcium levels. RBL-2H3 and BMMCs were sensitized with anti-DNP IgE. After incubation overnight, the cells were preincubated with Fluo-3/AM for 1 h, pretreated with or without drugs including SG-SP1, GA, and BAPTA for 1 h, and then challenged with DNP-HSA. Intracellular calcium was measured every 1 min for 5 min using a fluorescent plate reader. AUC was calculated over 5 min. BAPTA-AM, a calcium chelator, was used as a positive control. Data were presented as the means ± SEM of three independent experiments. *, **, and *** indicate statistically significant differences from the DNP-HSA-challenged group at a value of *p* < 0.05, 0.01, and 0.001, respectively.

Calcium acts as a major secondary messenger in intracellular mast cell signaling. A rise in intracellular calcium levels was rapidly induced by antigen-IgE cross-linking, which triggered mast cell degranulation and expression of inflammatory cytokines (20). Inositol 1,4,5-trisphosphate (IP_3_) generated by phospholipase C (PLC)γ binds to the IP_3_ receptor on the endoplasmic reticulum (ER) membrane, from which calcium is released; the influx of extracellular calcium is caused by calcium depletion signaling to the ER (21). In our experiments, intracellular calcium levels were elevated to a minute of treatment for antigen challenge and were reduced by SG-SP1 (Figure 4). BAPTA-AM, a calcium chelator, was used as a positive control.

### Effects of SG-SP1 on the Activation of FcεRI-Mediated Signaling Proteins in Mast Cells

**Figure 5:**
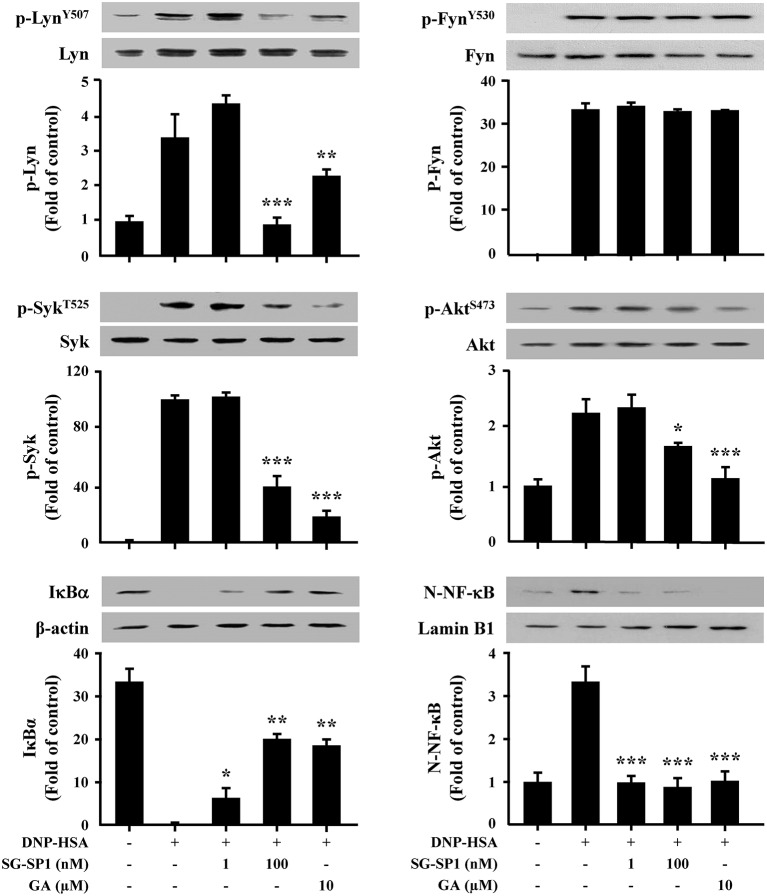
Effects of SG-SP1 on activation of NF-κB, and signaling proteins in mast cells. RBL-2H3 were sensitized with anti-DNP IgE. After incubation overnight, the cells were pretreated with or without SG-SP1 and GA for 1 h and then challenged with DNP-HSA. Activation of NF-κB and signaling proteins were assayed by Western blot (N-: nuclear, p-: phosphorylated). Lamin B1, β-actin, and total form of Lyn, Fyn, Syk, and Akt were used as loading controls. The band intensity was quantified and presented as the means ± SEM of three independent experiments. *, **, and *** indicate statistically significant differences from the DNP-HSA-challenged group at a value of *p* < 0.05, 0.01, and 0.001, respectively.

IgE-dependent mast cell activation leads to the secretion of three classes of mediators; degranulation results in secretion of preformed mediators that are stored in the cells' cytoplasmic granules, pro-inflammatory lipid mediators are synthesized *de novo*, and growth factors, cytokines, and chemokines are synthesized and secreted (4). To determine the mechanism of SG-SP1 on suppression of expression of inflammatory cytokines and degranulation, we investigated the effects of SG-SP1 on the activation of Lyn, Syk, and Akt, representative signaling proteins in mast cells. Phosphorylations of Lyn, Fyn, and Syk (5 min), and Akt (30 min) were increased by DNP-HSA stimulation. We found that SG-SP1 reduced phosphorylation of Lyn, Syk, and Akt. On the contrary, SG-SP1 did not alter the phosphorylation of Fyn (Figure 5).

In addition, NF-κB plays as a major transcription factor regulating the expression of inflammatory cytokines. NF-κB is translocated into the nucleus following degradation of IκBα (22). Our results presented that IκBα in the cytoplasm disappeared, whereas NF-κB in the nucleus increased after treatment with DNP-HSA. SG-SP1 inhibited NF-κB activation by obstructing IκBα degradation (Figure 5).

### Affinity Analysis of SG-SP1 to FcεRIα

**Figure 6:**
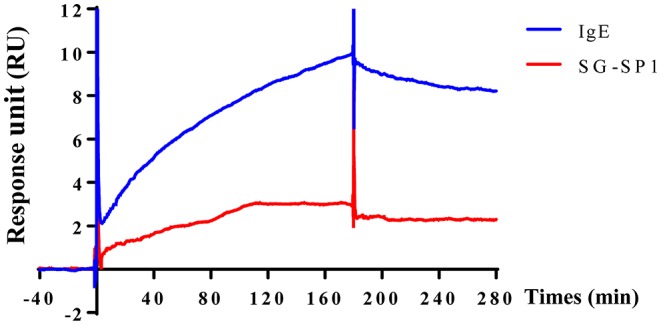
Surface plasmon resonance analysis of SG-SP1 binding to FcεRIα. SPR was performed using the CM5 chip containing ~1,300 response unit FcεRIα. SG-SP1 (100 μM) was diluted in HBS-EP buffer and flowed at a rate of 30 μL/mL for up to 300 s. SPR sensorgram showed the binding reaction of SG-SP1 like IgE (100 μM). SPR characterization of SG-SP1 interaction with FcεRIα compared to IgE, as a positive control.

To visualize the crosslinking of IgE or SG-SP1 on FcεRIα, SPR experiment was performed. As mentioned above, IgE-binding region is α subunit in FcεRI. For the SPR experiment, recombinant FcεRIα was coupled with CM5 sensor chip. Binding affinity with SG-SP1 to FcεRIα was comparatively analyzed compared to that of IgE, a positive control. The graph shows the interactions of compounds with FcεRIα (Figure 6). The response units obtained from the affinity analysis were elevated to both SG-SP1 and IgE. The theoretical prediction of SG-SP1 as a FcεRIα binding protein is supported by these results.

### Effects of SG-SP1 on Systemic and Local Anaphylaxis

**Figure 7:**
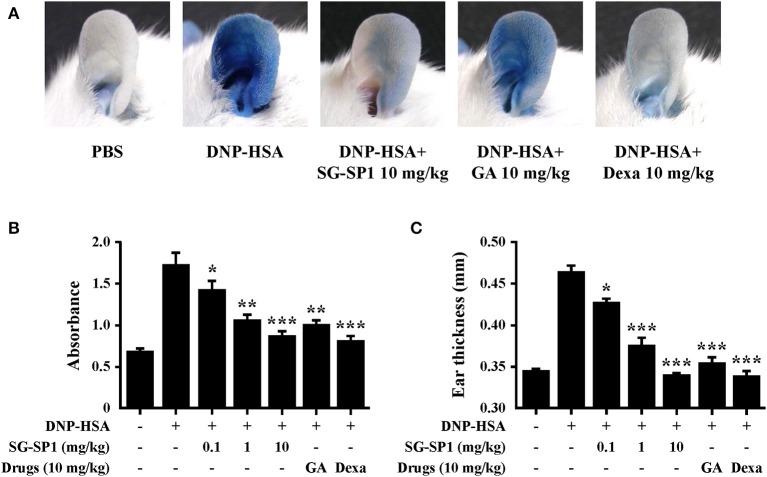
Effects of SG-SP1 on IgE-mediated PCA. Induction of PCA and oral administration of drugs including SG-SP1, GA, and Dexa were described in the section Materials and methods. **(A)** A representative image of pigmentation in each group. **(B)** Dye was extracted and then detected by spectrophotometry. **(C)** The thickness of both ears was measured 30 min after injection of DNP-HSA and 4% Evans blue (1:1) mixture. Data were presented as the means ± SEM (*n* = 5/group) of two independent experiments. *, **, and *** indicate statistically significant differences from the vehicle group at a value of *p* < 0.05, 0.01, and 0.001, respectively.

**Figure 8:**
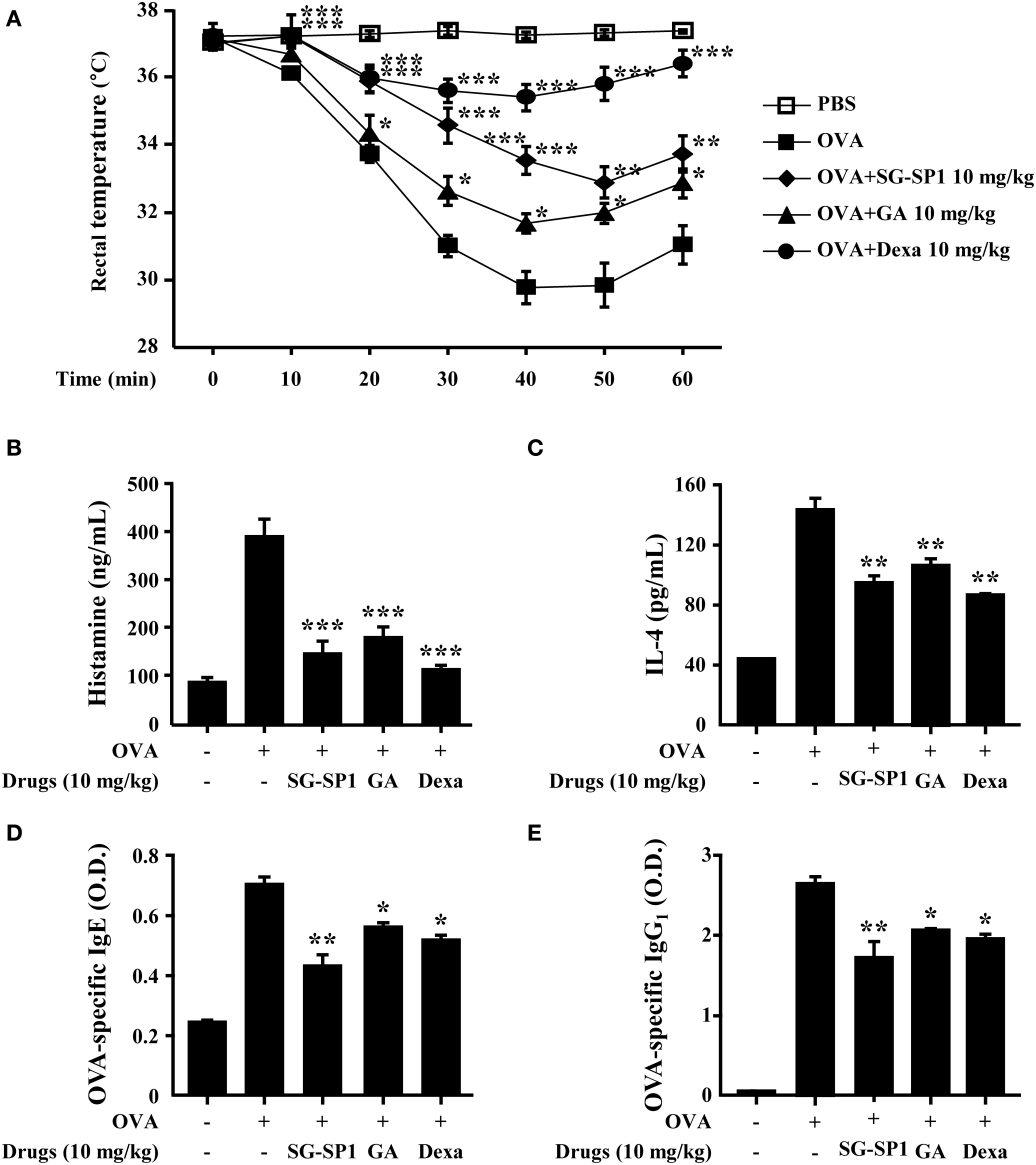
Effects of SG-SP1 on OVA-induced systemic anaphylaxis. Induction of systemic anaphylaxis and oral administration of drugs including SG-SP1, GA, and Dexa were described in the section Materials and Methods. **(A)** Rectal temperatures were measured every 10 min for 1 h. Blood was obtained from the abdominal vein of each mouse to measure serum histamine, IL-4, OVA-specific IgE, and OVA-specific IgG_1_ levels. **(B)** Histamine levels were assayed using an *o*-phthaldialdehyde spectrofluorometric procedure. **(C–E)** IL-4, OVA-specific IgE, and OVA-specific IgG_1_ levels were measured by ELISA. Data were presented as the means ± SEM (*n* = 5/group) of two independent experiments. *, **, and *** indicate statistically significant differences from the vehicle group at a value of *p* < 0.05, 0.01, and 0.001, respectively.

PCA is one of the most widely used animal models to demonstrate local anaphylaxis (8). After an injection of an antigen-Evans blue mixture, a single blue spot developed at the sensitized site with antigen-specific IgE because of enhanced vascular permeability. SG-SP1 alleviated the size and lightened the color of the spot on a dose-dependent manner (Figures 7A,B). Increased vascular permeability also induced ear swelling, which was reduced by SG-SP1 (Figure 7C). To investigate the inhibitory effects of SG-SP1 on immediate-type hypersensitivity, OVA-induced systemic anaphylaxis model was adopted. This model is useful for studying anti-allergic inflammatory activities of therapeutic candidates (8). Mice sensitized with OVA mixture experience anaphylaxis after additional challenge to OVA. The rectal temperature of the mice in the vehicle group decreased over 40–50 min, whereas the serum histamine level elevated approximately 4 folds. Oral administration of SG-SP1 moderated hypothermia and reduced serum histamine release (Figures 8A,B). Serum levels of IL-4, OVA-specific IgE, and IgG_1_ were increased with OVA challenge and were decreased by SG-SP1 (Figures 8C–E). SG-SP1 showed more powerful suppression of both local and systemic anaphylaxis than GA did and similar inhibition in a PCA model with Dexa, a positive control drug. All animals appeared to be in healthy condition, and no abnormal symptoms were observed in body weight and food intake by SG-SP1 throughout the administration period.

## Discussion

Diverse environmental and genetic factors are associated with the development of allergies (1). Asthma and allergic diseases are major public health problems affecting more than 300 million people worldwide (23, 24). Allergies can result in death as a result of respiratory obstruction and anaphylactic shock. Asthma-related deaths and emergency department visits for food-induced anaphylaxis have risen steadily over the past couple of decades (25, 26). Anti-histamines and NSAIDs are prescribed for mild-to-moderate allergic symptoms, and glucocorticoids are used at doses that correspond with the symptom severity (27, 28). Despite advances in the management guidelines of allergies, therapeutic alternatives remain unmet because current drugs act as palliatives. Chronic use of glucocorticoids is limited because of its various adverse effects including bruising, muscle weakness, weight gain, skin changes, sleep disturbances, and pathologic fractures (29).

Natural products-originated compounds have been considered safe sources of new drug candidates from decades (30). These compounds could be ameliorated by chemical modifications (31). Epigallocatechin gallate (EGCG), a major component of green tea, is known to have many biologic activities including anti-oxidation, anti-inflammation, and anti-bacterial/viral infections (32–37). GA, a part of EGCG, was shown to have inhibitory effects on mast cell-mediated allergic inflammation in our previous studies, and we have generated various derivatives of GA to improve its pharmacological effectiveness (10, 11). As mentioned above, to strengthen the effectiveness of GA, SG-SP1 was designed from GA (12). In this study, we aimed to compare the anti-allergic effects of SG-SP1 with those of GA.

Mast cells are known to be associated with immediate-type hypersensitivity through the release of allergic mediators and cytokines (38). To examine how SG-SP1 hinders allergic inflammation, we performed an *in vitro* assay for degranulation and cytokine expression in mast cells. Mast cells are key players in IgE-mediated allergic responses because they release allergic mediators such as histamine, lipid-derived mediators, chemokines, cytokines, and growth factors (39). Therefore, mast cells could be a proper target for the development of therapeutic agents for allergic diseases. Histamine from mast cells results in representative allergic symptoms such as edema, warmth, and erythema (38). Inflammatory cytokines promote and sustain chronic allergy. TNF-α induces an adaptive immune response by activating NF-κB and stimulating the migration, maturation, and differentiation of immune cells (40, 41). IL-1β exacerbates autoimmune and allergic diseases including atopic dermatitis, contact hypersensitivity, and bronchial asthma (42). IL-4, which causes plasma B cells to generate IgE, is necessary for an allergic response (43). SG-SP1 reduced the release of histamine and β-hexosaminidase, as well as the expression of TNF-α, IL-1β, IL-4, and IL-6. Based on these results, we determined that SG-SP1 could be beneficial to the treatment of allergic diseases.

Mast cell signaling has been researched in detail. Activation of FcεRI results in phosphorylation of Lyn and Syk, which stimulate Akt and PLCγ (4). PLCγ is the key signaling molecule that utilizes the conversion to phosphatidylinositol bisphosphate (PIP_2_) to second messengers, IP_3_ and diacylglycerol (44). After IP_3_ binds to its receptors on the surface of the ER, calcium stored in the ER is released into the cytoplasm. Signaling in response to calcium depletion in the ER triggers the rapid influx of extracellular calcium (21). Calcium plays a role of an important secondary messenger in mast cell signaling. The increase in intracellular calcium levels induces exocytosis, as well as transcription of histamine and inflammatory cytokine genes (45–47). Our results showed that SG-SP1 reduced calcium influx. Therefore, SG-SP1 demonstrated inhibition of mast cell degranulation through calcium blockade and the low level of histamine that results from blocked calcium diminishes the allergic reaction.

To evaluate the anti-allergic mechanisms of SG-SP1, we examined the FcεRI-mediated downstream signaling pathway. Lyn, Fyn, Syk, and Akt were chosen as representative signaling proteins, and activations of these proteins were decreased by SG-SP1. Especially, Lyn, a Src family kinase, is an important regulator of allergic diseases such as asthma and psoriasis because it binds to FcεRI directly (48). As additional study, to determine whether SG-SP1 binds to FcεRIα, IgE-binding region, and where it combines, SPR experiment was performed. We found that both IgE and SG-SP1 bound to FcεRIα. In our experiment, mast cells were sensitized with anti-DNP IgE before treatment of SG-SP1. This finding recapitulates that IgE binds to FcεRIα first, and then SG-SP1 binds to another site. Thus, we assumed that SG-SP1 suppresses mast cell signaling *via* binding to FcεRIα at a different region to IgE. These results convinced us that SG-SP1 inhibits the signaling cascade in mast cells by direct binding to FcεRI. It should be discussed the role of Fyn during the activation of mast cells. Several report showed that Fyn is vital to mast cell granule trafficking and degranulation (49, 50). On the other hand, Barbu et al. showed the limited contribution of Fyn in mast cells (51). In our preliminary experiments, SG-SP1 did not alter IgE-stimulated phosphorylation of Fyn. Therefore, we focused on the Lyn activation and downstream signaling pathway in this study. The exact role of Lyn and Fyn in mast cell activation is still debating. However, at least in our experimental condition, SG-SP1 reduced Lyn but not Fyn activation.

We investigated the biologic effects of SG-SP1 using systemic and local anaphylaxis models. These anaphylactic reactions are related to T helper (Th) 2 responses (52). Mice sensitized with OVA and alum adjuvants showed increased production of Th2 cytokines, especially IL-4, OVA-specific IgE, and IgG_1_. IL-4 released from antigen-presenting cells differentiates naive T cells into Th2 cells, which stimulate B cells to produce IgE and IgG_1_ that bind with FcεRI (43). Following challenge to OVA, a sudden increase in serum histamine levels contributed the hypothermia in mice (53). Another model, PCA is associated primarily with mast cell degranulation. Pigmentation and ear swelling are caused by histamine, because it enhances vascular permeability (43). We propose that SG-SP1 alleviated anaphylactic symptoms by inhibiting not only the release of histamine from mast cells but also the production of IgE and IgG_1_ *via* reduced IL-4 expression. There is an evidence that IL-4 plays a critical role in allergic sensitization through the production of IgE and IgG_1_. As the expression of IgE and IgG_1_ was impaired, allergic hypothermia was not observed in IL-4 deficient mice (54). SG-SP1 showed greater suppressive effects of both animal models than those of GA and inhibition equal to that of Dexa in animal models.

Although there have been reports on the anti-allergic effects of GA, its inhibitory mechanism has not been elucidated. Either, the new derivatives were made by supplementing the low metabolic stability of GA. SG-SP1 is designed as an analog based on GA. In the present study, both degranulation of mast cells and expression of inflammatory cytokines were suppressed by SG-SP1 and it was associated with the reduction of intracellular calcium levels and NF-κB activation. Mechanically, SG-SP1 inhibited the activation of Lyn/Syk pathway and its downstream signaling molecules to inhibit secretion of allergic mediators. The inhibitory effects result from SG-SP1 binding to FcεRIα, because SG-SP1 blocked mast cell signaling from Lyn. Thus, we confirmed that both IgE and SG-SP1 bound to FcεRIα. Therefore, additional studies might be needed to determine the exact binding region of SG-SP1, and to establish what mechanism is effective in inhibiting it after binding. In addition, the inhibitory effects of this mechanism were confirmed in animal models, SG-SP1 reduced both systemic and local anaphylaxis by inhibiting the release of allergic mediators. Nevertheless, results of this study provided conclusive evidence that SG-SP1 could be a novel therapeutic candidate to treat the allergic disease.

## Data Availability Statement

The datasets generated for this study are available on request to the corresponding author.

## Ethics Statement

The animal study was reviewed and approved by Institutional Animal Care and Use Committee of Kyungpook National University.

## Author Contributions

M-JK, I-GJ, JS, and XF carried out the major experiments and drafted the manuscript. HY, SL, and WK supported in the study design, reviewed the protocol, and participated to interpret the primary outcome. YJ, S-YS, and S-HK supervised the research and co-wrote the manuscript.

### Conflict of Interest

I-GJ was employed by the company ILDONG Pharmaceutical Co. Ltd. JS and HY were employed by the company Daegu-Gyeongbuk Medical Innovation Foundation. The remaining authors declare that the research was conducted in the absence of any commercial or financial relationships that could be construed as a potential conflict of interest.

## Abbreviations

Dexa: dexamethasone
DMSO: dimethyl sulfoxide
EGCG: epigallocatechin gallate
ER: endoplasmic reticulum
FcεRI: the high affinity IgE receptor
GA: gallic acid
HRMS: high-resolution mass spectra
IP_3_: inositol 1,4,5-trisphosphate
MTT: 3-(4,5-dimethylthiazol-2-yl)-2,5-diphenyltetrazolium bromide
NSAIDs: nonsteroidal anti-inflammatory drugs
OVA: ovalbumin
PCA: passive cutaneous anaphylaxis
PE: phycoerythrin
PLC: phospholipase C
qPCR: quantitative real-time polymerase chain reaction
RBL: rat basophilic leukemia
RPMCs: rat peritoneal mast cells
BMMCs: bone marrow-derived mast cells
SNAREs: soluble *N*-ethylmaleimide attachment protein receptors.
