# Supplementary material for: SG-SP1 Suppresses Mast Cell-Mediated Allergic Inflammation via Inhibition of FcεRI Signaling
Source: Front Immunol. 2020 Jan 28;11:50. doi: 10.3389/fimmu.2020.00050 (PMC6998798; doi:10.3389/fimmu.2020.00050)
Supplement: Supplementary file 1 [file Data_Sheet_1.docx]

**SG-SP1 Suppresses Mast Cell-Mediated Allergic Inflammation *via* Inhibition of FcεRI Signaling**

**Supplementary Figure S1.** Effects of SG-SP1 on cell viability.

**
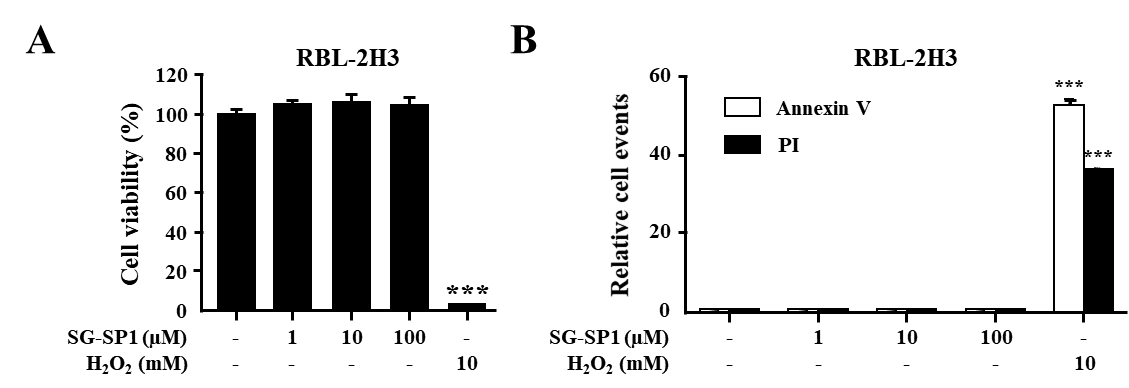
**

(A) RBL-2H3 (3×10^4^/well) were pretreated with or without SG-SP1 for 24 h and then incubated with 1 mg/mL MTT for 2 h. Hydrogen peroxide was used as a positive control. Absorbance intensities were determined using a spectrophotometry. (B) RBL-2H3 (5×10^5^/well) were pretreated with or without SG-SP1 for 24 h and then incubated with Annexin V and PI for 20 min. The fluorochrome was analyzed using a flow cytometer. Data were presented as the means ± SEM of three independent experiments. *** indicates statistically significant differences from untreated control at a value of *p* < 0.001.**Supplementary Table S1. Primers used in qPCR analysis.**

| Gene | Primer sequence | GenBank accession gene number |
| --- | --- | --- |
| rβ-actin | GGACTTCGAGCAAGAGATGG AGCACTGTGTTGGCGTACAG | NM_031144.3 |
| rTNF-α | TCCCAAATGGGCTCCCTCTC  AAATGGCAAACCGGCTGACG | NM_012675.3 |
| rIL-1β | GCTGTGGCAGCTACCTATGTCTTG  AGGTCGTCATCATCCCACGAG | NM_031512.2 |
| rIL-4 | TGCACCGAGATGTTTGTACCAGA  TTGCGAAGCACCCTGGAAG | NM_201270.1 |
| rIL-6 | TGTGCAATGGCAATTCTGAT  GAGCATTGGAAGTTGGGGTA | NM_012589.2 |
| mβ-actin | ACCCTAAGGCCAACCGTGAA  ATGGCGTGAGGGAGAGCATAG | NM_007393.5 |
| mTNF-α | CCCCAAAGGGATGAGAAGTTC  GGCTTGTCACTCGAATTTTGAGA | NM_013693.3 |
| mIL-1β | ATAACCTGCTGGTGTGTGAC  AGGTGCTGATGTACCAGTTG | NM_008361.4 |
| mIL-4 | ATCATCGGCATTTTGAACGAGGTC  ACCTTGGAAGCCCTACAGACGA | NM_021283.2 |
| mIL-6 | TCCAGTTGCCTTCTTGGGAC  GGTCTGTTGGGAGTGGTATC | NM_001314054.1 |
